# Evaluation of the Behavior of Two CAD/CAM Fiber-Reinforced Composite Dental Materials by Immersion Tests

**DOI:** 10.3390/ma14237185

**Published:** 2021-11-25

**Authors:** Farah Bechir, Simona Maria Bataga, Adrian Tohati, Elena Ungureanu, Cosmin Mihai Cotrut, Edwin Sever Bechir, Mircea Suciu, Diana Maria Vranceanu

**Affiliations:** 1 Doctoral School of Medicine and Pharmacy, George Emil Palade University of Medicine, Pharmacy, Science, and Technology of Targu Mures, 38 Gh. Marinescu Str., 540142 Targu Mures, Romania; farah.bechir@yahoo.com; 2 Faculty of Medicine, George Emil Palade University of Medicine, Pharmacy, Science, and Technology of Targu Mures, 38 Gh. Marinescu Str., 540142 Targu Mures, Romania; simonabataga@yahoo.com; 3 Faculty of Dental Medicine, George Emil Palade University of Medicine, Pharmacy, Science, and Technology of Targu Mures, 38 Gh. Marinescu Str., 540142 Targu Mures, Romania; mircea.suciu@umfst.ro; 4 Faculty of Materials Science and Engineering, Politehnica University of Bucharest, 313 Splaiul Independentei, 060042 Bucharest, Romania; ungureanu.elena14@yahoo.com (E.U.); cosmin.cotrut@upb.ro (C.M.C.); diana.vranceanu@upb.ro (D.M.V.)

**Keywords:** CAD/CAM, various pH values, GERD, fiber-reinforced composites, biomaterials, dentistry

## Abstract

Fiber-reinforced composites are used as restorative materials for prosthetic oral rehabilitation. Gastroesophageal reflux disease (GERD) is an accustomed affection with various oral manifestations. This study aimed to evaluate the behavior of two high-performance CAD/CAM milled reinforced composites (Trinia™, TriLor) in artificial saliva at different pH levels through immersion tests, and to determine if changes in mass or surface morphology at variable pH, specific for patients affected by GERD, appear. After investigating the elemental composition and surface morphology, the specimens were immersed in Carter Brugirard artificial saliva for 21 days at different pH values (5.7, 7.6, and varying the pH from 5.7 to 3). The values of the weighed masses during the immersion tests were statistically processed in terms of mean and standard deviation. Results suggested that irrespective of the medium pH, the two composites presented a similar mass variation in the range of −0.18 (±0.01)–1.82 (±0.02) mg after immersion, suggesting their stability when in contact with artificial saliva, an aspect which was also highlighted by scanning electron microscope (SEM) analysis performed on the immersed surfaces. Novel composite biomaterials can be a proper alternative for metal alloys used for prosthetic frameworks in patients suffering from GERD.

## 1. Introduction

Although composites were first used by early Egyptians and Mesopotamian settlers back in 1500 BC [1], they were introduced as dental restorative materials during the late 1950s. Since then, we have witnessed further improvement by developing enhanced composites with long-term durability [2]. Synthetic polymeric materials used as dental biomaterials are compounds used for the treatment, regeneration, or replacement of tissues, organs, or functions of the body, due to their physical, chemical, and mechanical properties [3,4]. Dental biopolymers used in the manufacturing of various prosthetic restorations represent the comprehensive class of biomaterials with large applications in dentistry [5]. 

Most fiber-reinforced composite (FRC) dental materials contain three distinct constituent parts: (1) the continuous phase (which is also named matrix), (2) the dispersed phase (formed by the fibers, generally glass fibers), and (3) the interphase area [6]. The matrix, which is the majority, takes over the external stress forces on its surface and transforms them at the level of the dispersed phase, which often plays the role of reinforcement material, giving greater strength to the composite material [7]. Theoretically, there is an infinite number of possible combinations between different materials, which can be used both as majority phases and as minority phases, in order to obtain a composite material with the desired properties. From a practical point of view, high-performance reinforced composite materials with use in various fields have been obtained, from the aerospace industry to the industry of materials used in medicine and dentistry [8]. 

Often, the aim of developing a new class of dental reinforced composites was to improve their mechanical strength, to prolong their use, and to acquire other important characteristics, such as specific weight and low cost. Thus, currently, the ratio of resistance/specific weight, and stiffness/specific weight is optimized [7,9]. Among the disadvantages of metals used in dentistry, the potential allergenicity, their weight, density, as well as their long processing time are included. The color of metal restorations has a particular aspect that is different from the gingiva and the teeth color shade. Additionally, metals have great strength, are harder than natural teeth, have a very good thermal conductivity, and inferior thermic insulation in comparison to the natural teeth [10]. Furthermore, free ions released in the oral cavity because of metal corrosion can cause problems regarding the function of the prosthetic restoration and can influence the biological response to the dental alloy [11]. For these reasons (the disputable aesthetics of dental metals and alloys, as well as their physical and chemical properties), the research for other types of dental materials, preferably metal-free, are sustained [12,13]. The benefits of metal-free biomaterials for dental restorations are represented by a more natural appearance without metallic elements, for an easier correction of the shade, to match to any tooth, to be highly attractive, and to look and feel like the natural teeth [14,15]. The metal-free prosthetic restorations present biocompatibility; therefore, the negative impact of metal on the patient’s body is eliminated [10]. 

Innovations in computer aided design and computer aided manufacture (CAD/CAM) have made the manufacture of new restorative and prosthetic materials possible [16].

Trinia™ (BICON, Boston, MA, USA) is a fiber-reinforced dental composite for permanent computer aided design and computer aided manufacturing (CAD/CAM) milled dental prosthetic rehabilitations, presented as discs and/or blocks, and it represents an alternative to conventional technological methods. It is a metal-free reinforced dental composite, formed of multidirectional interlacing of a fiberglass and resin matrix in several layers, that has high flexural strength and a flexural modulus of elasticity similar to dentin [17,18,19]. The properties and versatility of Trinia™ reinforced composites permit their use for permanent dental prosthetic restorations because of their relatively simple design and manufacturing by using CAD techniques. Trinia™ can be machined with customary wet- or dry-milling machine systems using nano-diamond burs. The flexural rigidity and the compression strength of Trinia™ are high despite the minimal CAD/CAM processing time. Trinia™ can be manufactured extra-orally as well as intra-orally and presents high comfort while wearing because of its light weight [20,21]. 

TriLor (Bioloren, Saronno, Italy) is a high-performance biocompatible techno-polymer, and its matrix is reinforced with multi-directional glass fibers. It is indicated for a large variety of permanent esthetic metal-free dental prosthetic restorations on implants, such as removable partial dentures, full arch implant supra-structures, and bridge frameworks. Because of its flexing and bending capacity under stress, TriLor represents an ideal milled composite for implant-supported restorations and creates resilient frames/substructures for zirconia, lithium disilicate, acrylics, and composites, due to its characteristics which resemblance natural bone [22,23,24]. 

Dental materials interact continuously with the oral environment and with the oral microbiome metabolism. These factors lead to a decreased life of prosthetic restorations used for the oral rehabilitation of patients [25,26]. The gastroesophageal reflux disease (GERD) is an accustomed affection, with approximately 50% of all adults reporting reflux symptoms at some time during their lives [27]. A lot of oral symptoms appear in GERD (i.e., oral dryness, glossalgia, dental erosion, inflammation of the oral mucosa) due to the acidic pH of oral fluids, increased by the acid reflux [28]. 

The evaluation models should be pertinent for the in vivo environment to assure that the dental materials will withstand in the oral environment without any depreciation [29,30].

Therefore, the aim of this study was to evaluate the behavior of two high-performance reinforced dental composites (Trinia^TM^ and TriLor) in artificial saliva at different pH levels through immersion tests and to determine if changes in mass or surface morphology at variable pH, specific for patients affected by GERD, appear.

## 2. Materials and Methods

### 2.1. Sample Preparation

The experimental samples used in this study were obtained using CAD/CAM technology. The trade name of the selected materials, as well as the manufacturer from which they were purchased along with the sample codification, are presented in Table 1. The samples were obtained in the form of discs with the following dimensions: diameter of 15 mm and a thickness of 5 mm.

### 2.2. Morphology and Chemical Composition 

The experimental samples presented in Table 1 were investigated in terms of elemental composition and surface morphology with a scanning electron microscope, equipped with an X-ray energy dispersive spectrometer (SEM-EDS, Phenom ProX, Phenom World, Eindhoven, The Netherlands). The surface morphology and elemental composition were analyzed before and after performing the immersion tests in artificial saliva with different pH values. 

### 2.3. Immersion Assay

**Figure 1:**
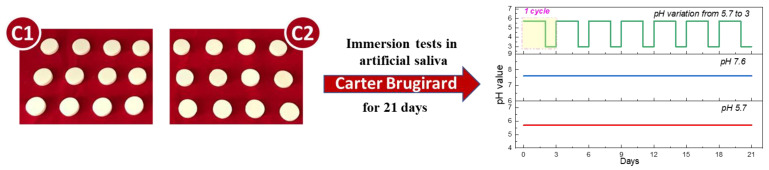
Images of the experimental samples (C1—Trinia^TM^ and C2—TriLor) and pH value of the artificial saliva.

The immersion tests represent a non-invasive, facile, and rapid method to evaluate a material behavior in a synthetic body fluid used as testing media. Because the composite biomaterials tested in this study are used in dentistry, artificial saliva was selected as the testing medium. Moreover, considering that not all patients exhibit similar salivary pH values due to their medical record or other factors, three pH values were selected to establish if the acidity of the artificial saliva influences the materials’ behavior and/or their stability. The pH values of the artificial saliva used to immerse the samples were selected as follows: 5.7, 7.6, and a varied pH (5.7–3). In the case of the varied pH, the samples were immersed for 2 days in pH 5.7 and on the third day, it was changed to pH 3 for one day, as can be observed in Figure 1. This cycle was repeated 7 times. 

Figure 1 presents the experimental samples of both materials, C1 and C2, used in this study and a schematic illustration of the pH selected for the artificial saliva. 

The immersion assays were carried out for 21 days at a constant temperature of 37 ± 0.1 °C by using a Memmert IF55 incubator (Memmert GmbH, Büchenbach, Germany) in Carter Brugirard artificial saliva with the chemical composition presented in Table 2. 

Prior to immersion, the containers in which the tests were performed were irradiated with a UV-C lamp (280–100 nm) (Segula BmbH, Horb am Neckar, Germany) to sterilize them, thus, preventing the formation of biofilms on their surface during the tests. 

The materials’ behavior in artificial saliva with different pH values was evaluated by monitoring their mass loss/gain at different time intervals (3, 7, 14, and 21 days) with a Kern ALT 100–5 am balance with an accuracy of 0.01 mg. The samples were removed from the medium at predetermined intervals and were cleaned with distilled water, dried in hot air, and subsequently dried for 5 h at a temperature of 80 °C in a Memmert UF55 oven (Memmert GmbH, Büchenbach, Germany). They were then kept in a desiccator until they were weighed [31]. During the tests, the artificial saliva media were changed periodically (every 2 days) to permanently ensure the ionic concentration of the solutions and also to prevent the formation of biofilms.

For immersion assay, a number of 4 specimens of each material and the mass measurements were repeated 5 times for each sample. The samples mass variation was calculated using the mathematical formula below:

Δm = m_f_ − m_i_ (mg) (1)

where:

- Δm represents the mass variation;
- m_f_ is the mass of the sample after immersion;
- m_i_ is the initial mass of the sample.

After monitoring the mass gain/loss, the samples were analyzed in terms of morphology by SEM, to establish if the pH value has an impact on the materials’ behavior.

## 3. Results

### 3.1. Characterization of Fiber-Reinforced Resin Materials before Immersion Tests

#### 3.1.1. Morphology

**Figure 2:**
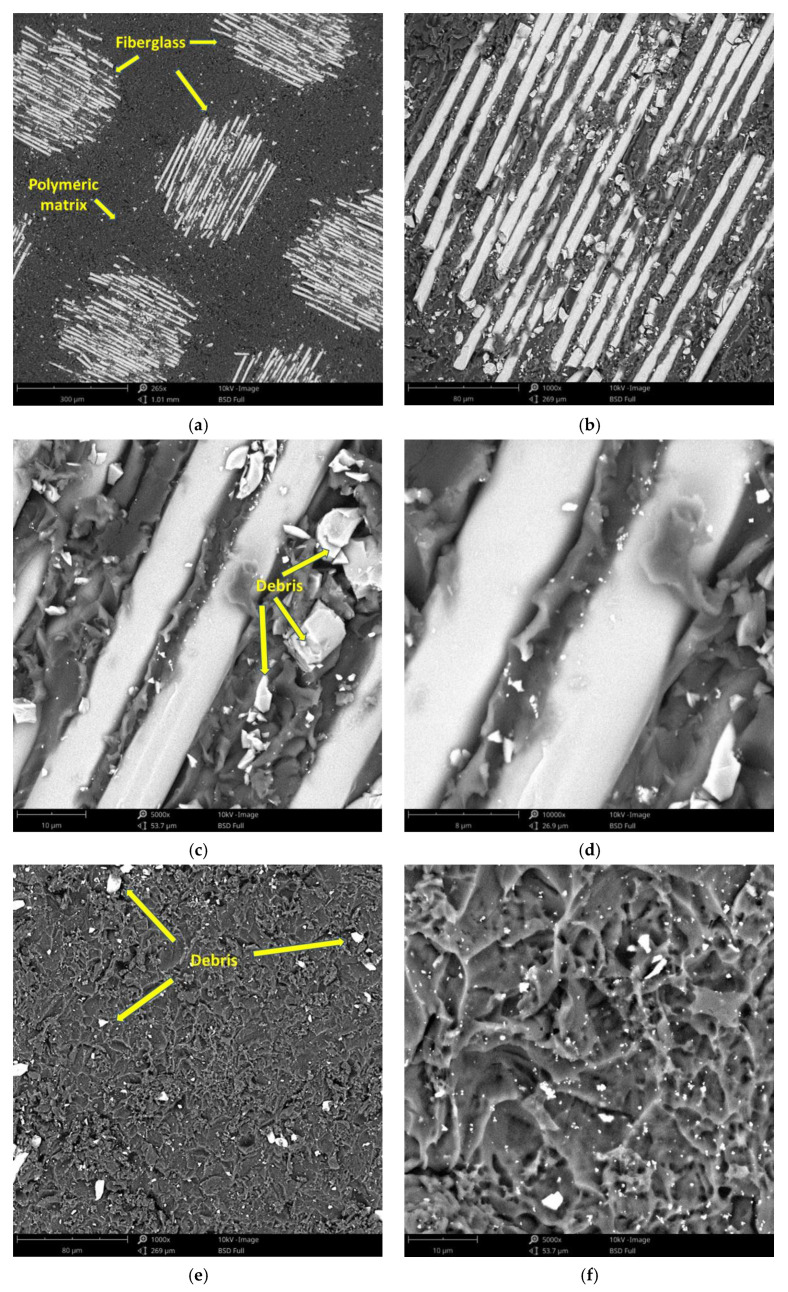
SEM images of sample C1 surfaces: (**a**) fiberglass at 265×; (**b**) fiberglass at 1000×; (**c**) fiberglass at 5000×; (**d**) fiberglass at 10,000×; (**e**) polymer matrix at 1000×; (**f**) polymer matrix at 5000×.

**Figure 3:**
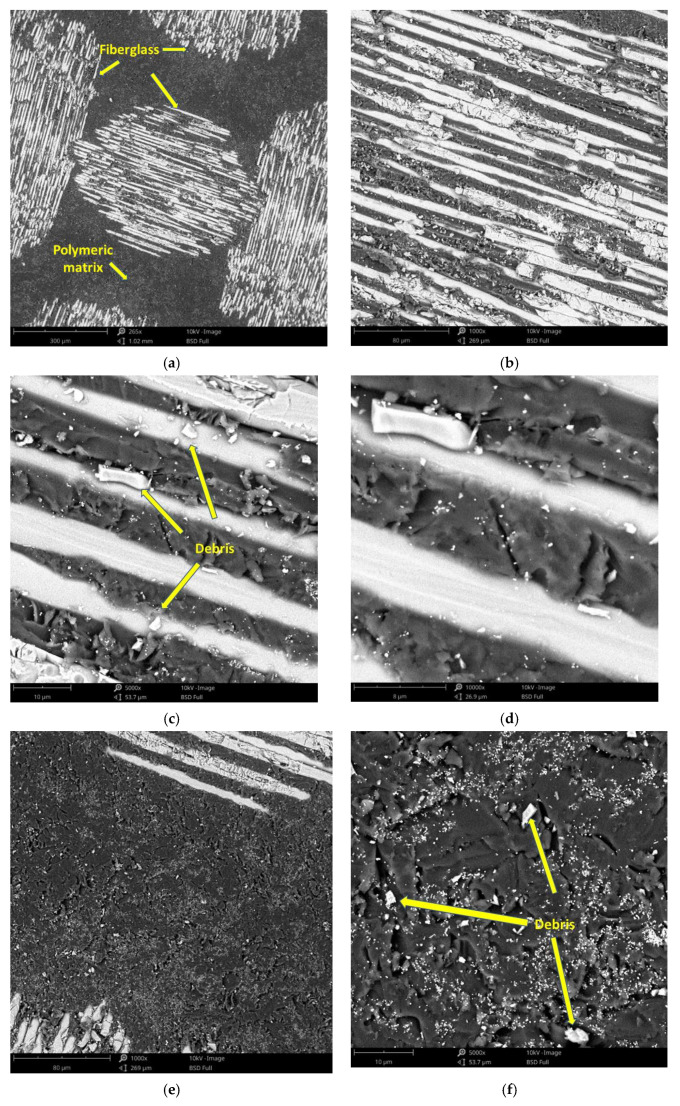
SEM images of C2 sample surfaces: (**a**) fiberglass at 265×; (**b**) fiberglass at 1000×; (**c**) fiberglass at 5000×; (**d**) fiberglass at 10,000×; (**e**) polymeric matrix at 1000×; (**f**) polymeric matrix at 5000×.

The surface morphology obtained by SEM of the two fiber-reinforced resin materials used in the study, C1 and C2, are presented in Figure 2 and Figure 3. Both materials consist of a polymer matrix of epoxy resin in which multidirectional oriented glass fibers are integrated. Thus, as can be seen in Figure 2 and Figure 3, both materials have reinforcing elements in the form of fibers, arranged differently, having a “textile fabric” appearance, fixed in epoxy resin.

Regarding material C1 (Figure 2), it can be observed that the reinforcing elements made of fiberglass appear to have a lower density compared to material C2 (Figure 3). 

In the SEM images of the material surfaces in Figure 2 and Figure 3, small fragments (debris) obtained following the milling process of the resin materials can be observed in both the reinforcing material (fiberglass) and the base material (polymeric matrix). The polymeric matrix of both resin materials present porosities of various shapes and sizes.

#### 3.1.2. Chemical Composition

**Figure 4:**
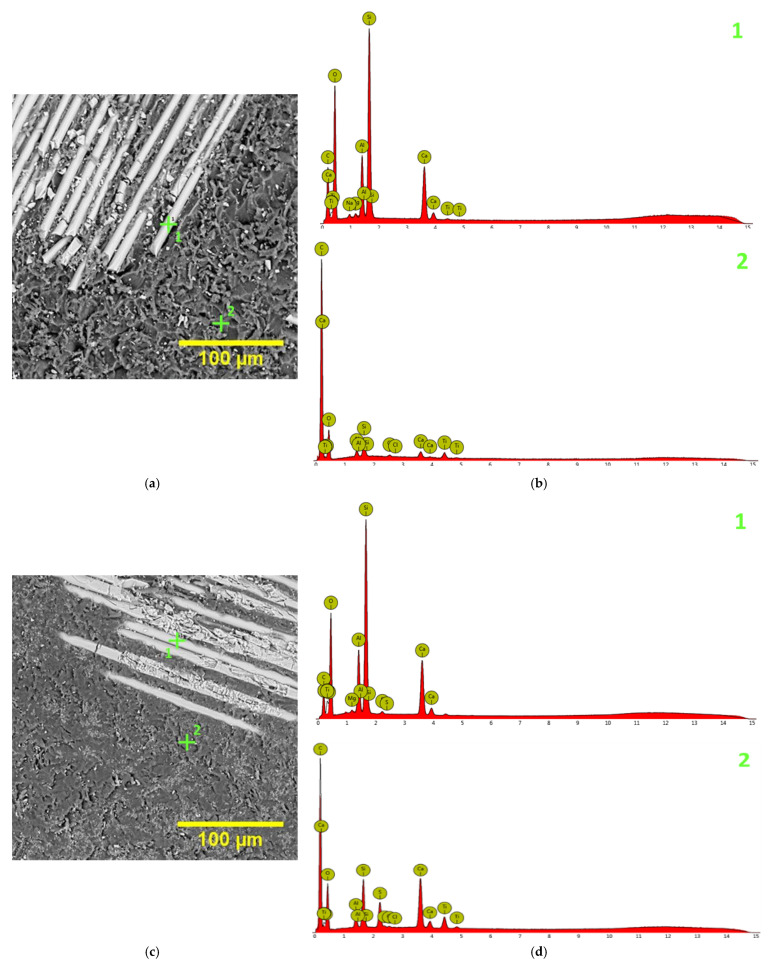
SEM images marking the points where the chemical composition was investigated (**a**,**c**) and the resulting EDS spectra (**b**,**d**).

The elemental composition of the materials used in this study, obtained using EDS in points depending on the phases/materials identified, is found in Table 3, while Figure 4 shows the SEM images in which the areas where the chemical composition was investigated are marked, alongside with the EDS spectra resulting for both C1 and C2 samples.

### 3.2. Immersion Assay in Artificial Saliva 

**Figure 5:**
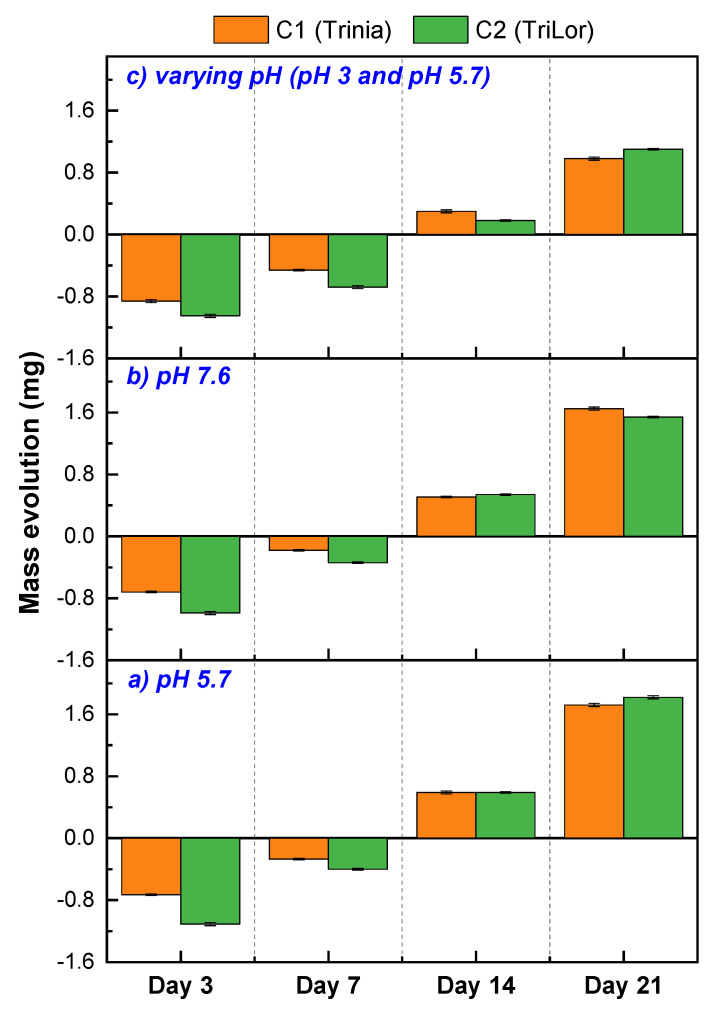
Evolution of the samples mass in artificial saliva: (**a**) of pH 5.7; (**b**) of pH 7.6; (**c**) of varied pH (5.7–3).

The evolution of the samples’ mass within the established time intervals (3, 7, 14 and 21 days) can be found in Table 4 for the samples tested in artificial saliva with different pH values. All the obtained results are also graphically represented in Figure 5.

Following the immersion tests, the mass of both studied composite materials shows a similar variation regardless of the pH of the artificial saliva used as a testing medium. Thus, after 3 and 7 days, the mass of the samples tends to decrease and starting with the 14th day it starts to increase, reaching its highest value after 21 days. However, it should be mentioned that the variation of the registered mass is not very large, being in the range of −0.18 (±0.01)–1.82 (±0.02) mg.

### 3.3. Surface Morphology after Immersion Tests in Artificial Saliva

**Figure 6:**
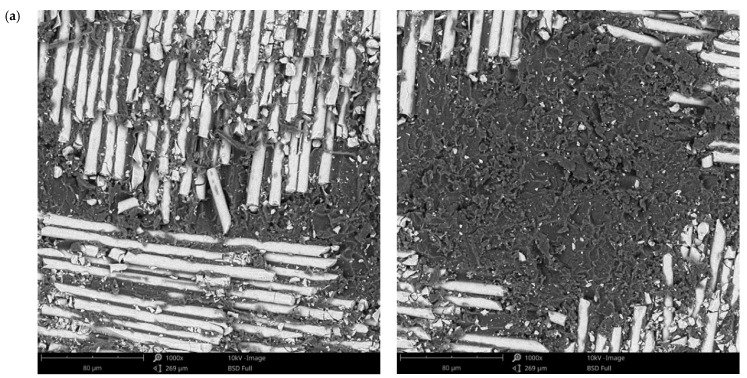

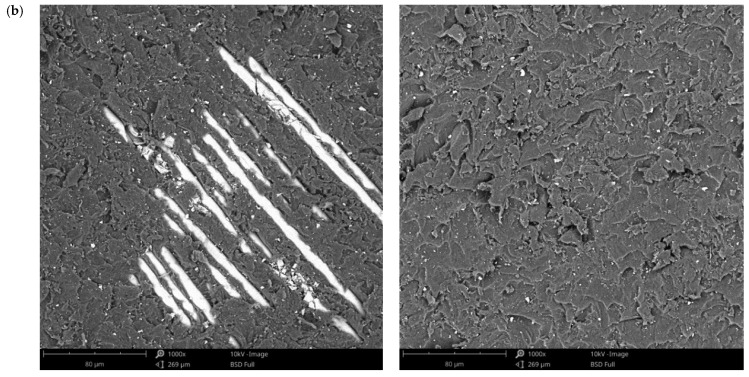
SEM images of the C1 sample surface after immersion tests at (**a**) 3 days and (**b**) 21 days in artificial saliva with pH 5.7 (1000×).

**Figure 7:**
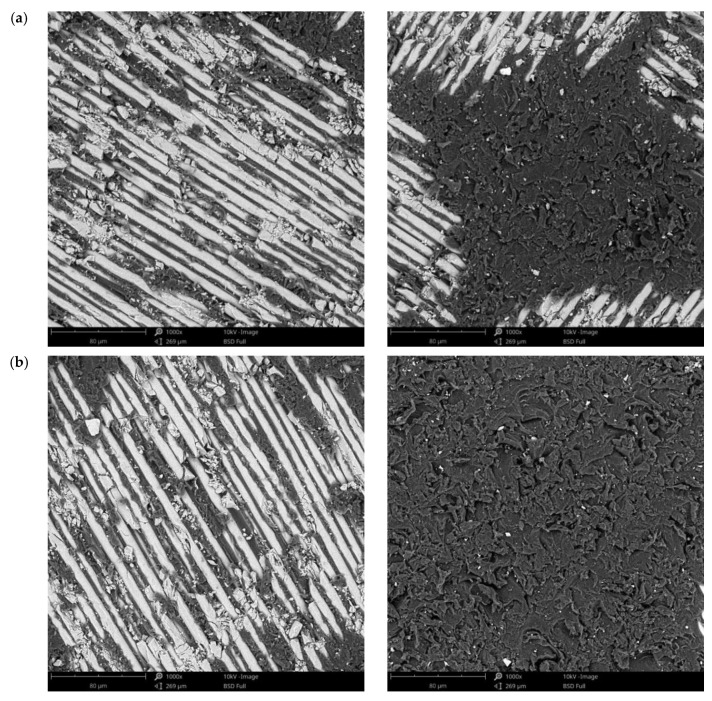
SEM images of the C1 sample surface after immersion tests at (**a**) 3 days and (**b**) 21 days in artificial saliva with pH 7.6 (1000×).

**Figure 8:**
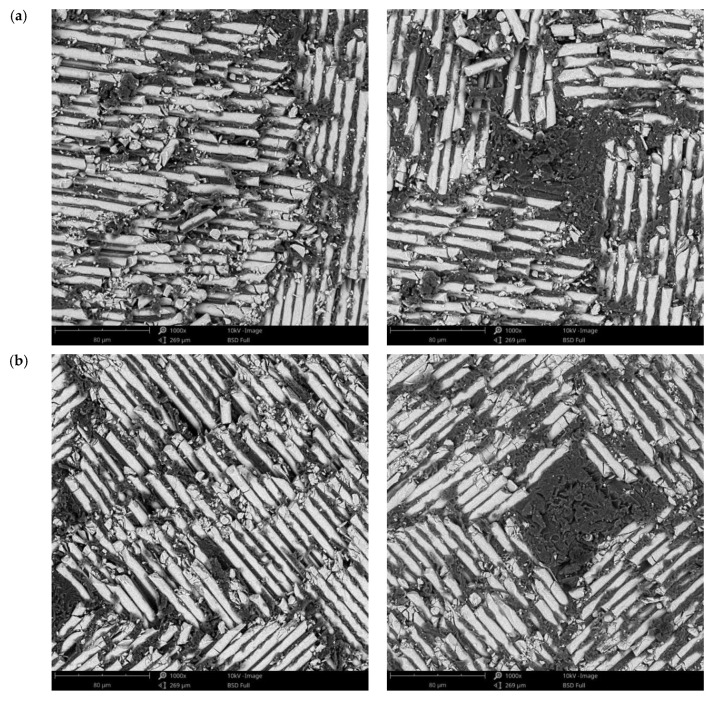
SEM images of the C1 sample surface after immersion tests at (**a**) 3 days and (**b**) 21 days in artificial saliva with varying pH (1000×).

**Figure 9:**
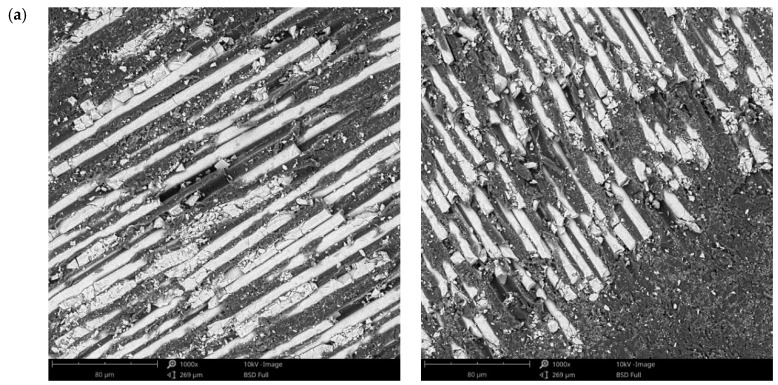

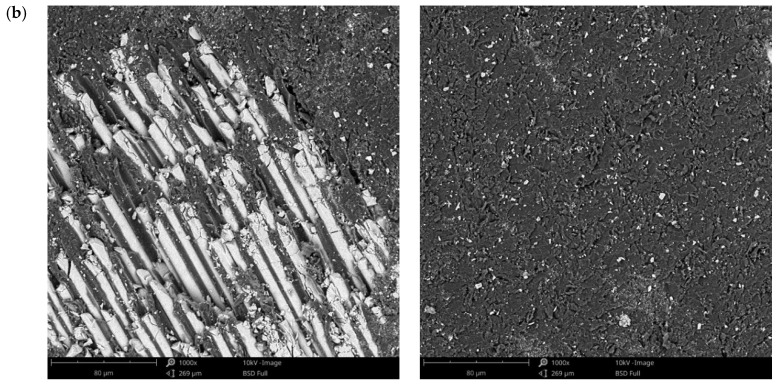
SEM images of the C2 sample surface after immersion tests at (**a**) 3 days and (**b**) 21 days in artificial saliva with pH 5.7 (1000×).

**Figure 10:**
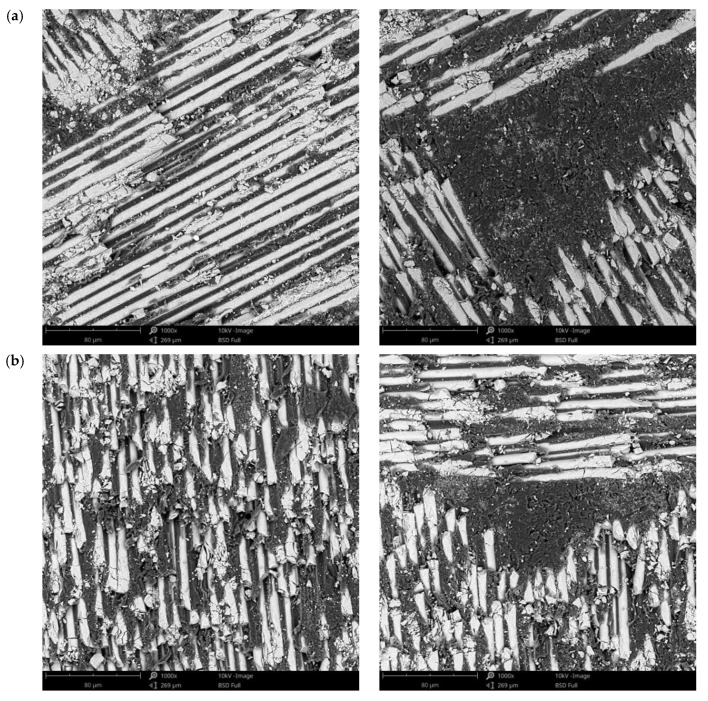
SEM images of the C2 sample surface after immersion tests at (**a**) 3 days and (**b**) 21 days in artificial saliva with pH 7.6 (1000×).

**Figure 11:**
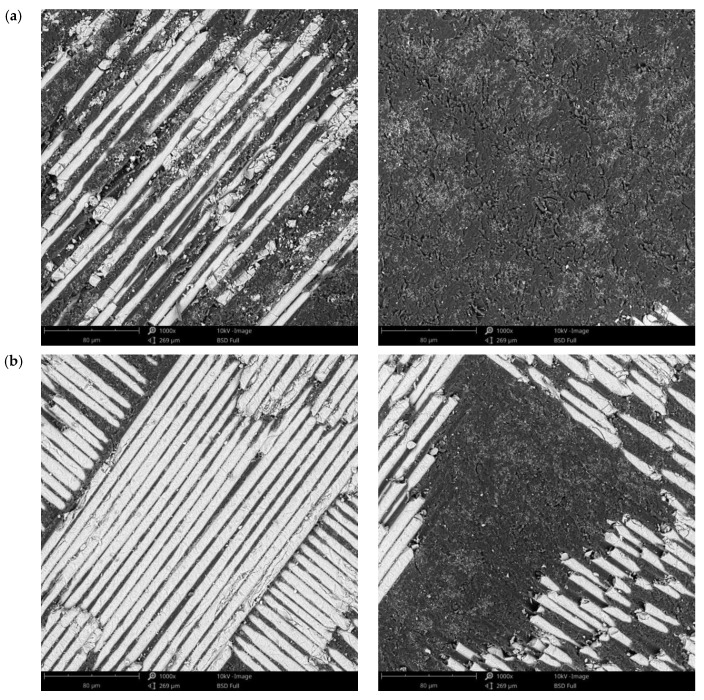
SEM images of the C2 sample surface after immersion tests at (**a**) 3 days and (**b**) 21 days in artificial saliva with varying pH (1000×).

For a detailed analysis of the samples’ surfaces after immersion tests, they were investigated by SEM. For these investigations, samples were selected after 3 and 21 days of immersion, for all pH types, and the images obtained are presented in Figure 6, Figure 7 and Figure 8 for C1, and Figure 9, Figure 10 and Figure 11 for C2. This selection considered the maximum mass variations of the samples following the immersion tests.

After a careful analysis of SEM images of the investigated surfaces of both composite materials before and after the immersion tests (studying both components, namely resin matrix and fiberglass), it can be stated that there are no notable differences and that the immersion tests did not induce morphological changes in any of the two composite materials regardless of the pH value used or the immersion period. 

## 4. Discussion

CAD/CAM technology eliminates dimensional changes during the laboratory manufacturing process. In order to achieve a high modulus of resilience and great flexural strength, composite resin-based materials were manufactured using this technology, with filler particles bonded to resin matrix [32]. 

In order to obtain optimal compatibility of filler particles with resin phase, various surface modifications techniques of filler particles have been suggested [33].

A reinforcement of resin materials with glass fibers significantly improves their mechanical and functional characteristics, as well as and biological tolerance, making them a viable solution for the prosthetic rehabilitation of patients [34].

Fiber-reinforced composite (FRC) dental materials have been specifically studied and advanced for over 25 years [35,36]. In FRC dental materials, the fibers are included particularly for their high specific modulus (stiffness and weight), as well as their specific strength and weight [7,37]. 

The surface morphology obtained by SEM of the two fiber-reinforced resin materials used in the study showed that Trinia^TM^ appears to have a lower density of reinforcing elements made of fiberglass, when compared to TriLor. This statement is closely correlated with the data from the literature but also the technical data provided by the manufacturer, which states that Trinia^TM^ (Bicon Dental Implants) has approximately 55% solid fiberglass [38], while for Trilor (Bioloren), the glass fiber is found in approximately 75% of the total volume [35,39,40].

With a flexural strength of 393 Mpa, Trinia’s application can be expanded into posterior regions of the mouth, with use in frameworks of fixed dental restorations and implant prostheses [41]. Trinia presents outstanding properties besides a high flexural strength, such as a flexural modulus of elasticity comparable to dentin and a high level of resilience. Trinia has been demonstrated to simulate the performance of Sharpey’s fibers due to its so-called buffering feature. Another feature of this FRC is the very low water adsorption, namely 0.03% [20].

A common concern regarding dental materials is their hydrolytic degradation due to the continuous exposure to the aqueous environment of the oral cavity [42].

Water sorption is realized by two means: adsorption and absorption. Adsorption is the attachment of molecules of a liquid or gas on the resin surface and absorption happens by penetration of liquid or gas within the mass of the composite resins [43]. Water sorption and hygroscopic expansion are among the physical properties of the composites. Water sorption may adversely affect the quality of the composite, thereby changing its efficiency as a restorative material [44].

Because water serves as a plasticizer, when absorbed by resins, it may cause discoloration, increased solubility, dimensional expansion and may affect their properties such as fatigue limit, transverse strength, and hardness. In addition, water sorption causes three-dimensional expansion, and this can lead to dimensional instability of the prostheses framework [45].

The oral environment can interfere with the water sorption of composite resins, due to its pH variation, either through acidic foods or organic acids (lactic and acetic acid) [43] or, in case of patients suffering from GERD, through the regurgitation of gastric juices into the oral cavity [46].

Water absorption and dimensional change of CAD/CAM polymers may be affected by variations in the composition aiming to improve the mechanical properties of these polymers. This phenomenon happens due to the fluid absorption in the polymer matrix, the highest moisture absorption generally occurring within the first few days, following a tendency to stabilize [47,48]. 

Based on the SEM images obtained after the immersion assay in our study, the weight loss registered in the first two monitoring periods (3 and 7 days) may be due to the loss on the surface of some residues/debris resulting from the milling process and the weight gain of the samples in the last periods of monitoring the samples’ masses (14 and 21 days) may be due to the infiltration of artificial saliva into the pores of the polymeric matrix and the crystallization of the salts inside them after drying. Both processes mentioned take place simultaneously but the first is predominant in the first part of the tests, while the second is during the second part.

Suzaki et al. [18] tested the water absorption of Trinia, a fiber-reinforced composite (everX posterior, GC) and a conventional composite (Beauti core flow paste, SHOFU) by immersing the samples in water at 37 ◦ C for 7 days. The lowest water absorption (4.7 ± 1.9 g/mm^3^) was found in Trinia out of all three materials. However, flexural strength significantly decreased from 24 h to 1 week, as opposed to the FS in everX posterior and Beauti core flow paste immersion groups. In Trinia and everX posterior groups, all specimens demonstrated incomplete fracture after the three-point bending test while in Beauti core flow paste group, all specimens demonstrated complete fracture. A smaller elastic modulus after 1 week was found, without any significant difference between the samples.

The highest impact on solubility found by Liebermann et al. [47] in their study on different computer-aided design and computer-aided manufacturing (CAD/CAM) polymers was for storage in physiological saliva. This result is probably due to the complex mixture of organic and inorganic components of natural saliva, which lead to a higher solubility rate than artificial saliva and especially NaCl and distilled water.

Gusmão et al. [43] studied the water sorption of nine specimens of nanoparticle, microhybrid, and nanohybrid composite resins stored in artificial saliva with pH 7.0 (control) and varying pH (4.3–7.0) for the test group. Weight gain for all the resins at all-time intervals was observed and the authors concluded that the degree of sorption is dependent upon storage time and composite chemical composition and was affected by the medium pH.

It is important to note that the use of artificial saliva simulates only the hydrolytic degradation of composite [49]. The rate of hydrolytic degradation depends on environmental factors (temperature, pH), storage time, and polymer properties, including resin matrix/filler particles proportion [47].

In his dissertation thesis, Abdallah [50] concludes that full-arch implant prostheses with Trinia framework are a viable option for fixed implant rehabilitation demonstrating damping capacity, adequate failure load values, and easy repairability.

Not many studies regarding the behavior of Trilor’s mechanical properties after immersion tests were found in the literature. In Ruschel et al. study [51], 10 prefabricated glass fiber posts (Exacto #3; Angelus, Londrina, PR, Brazil), 10 diagonally and 10 vertically CAD/CAM milled posts (Trilor, Bioloren) were evaluated using laser confocal microscopy and a parallel fiber distribution of glass fibers was observed for the prefabricated post, while the diagonally milled post presented multi-direction fiber distribution and the vertically milled one showed transversal parallel fiber distribution. The posts were not previously aged and authors concluded that prefabricated glass fiber posts presented higher flexural strength and modulus, as well as higher superficial roughness, in comparison to Trilor CAD/CAM milled posts.

After Scribante [37] and Sfondrini [52], the limiting clinical use of FRCs, despite the numerous studies carried out in vitro, is represented by the absence of long-term clinical studies regarding the efficiency of these dental materials.

Although Musanje concluded that the best method of simulating oral conditions in vitro is the use of artificial saliva at 37 °C [53] and aging the composites in artificial saliva is considered to simulate the clinical situation [49], this type of medium does not replicate the properties and the mechanical and chemical effects that physiological saliva has on composite materials. The immersion tests performed in our study were limited by the difficulty to mimic the harsh oral environment conditions, including temperature variation and acids present in food. 

Laboratory tests provide only some evidence of reliable values, and supplementary clinical studies are required to validate the obtained results and to improve the quality and the properties of FRC dental materials [47].

In advance of long-term clinical trials, in vitro testing of any new biomaterial is required to initially validate the manufacturer’s claims and provide initial predictors for restoration success [54]. For the advancement in the dental materials used for the achievement of prosthetic restorations, research for superior biomaterials should continue [5,55]. 

## 5. Conclusions

The results of our study reveal the necessity for the preclinical evaluation of novel metal-free biomaterials to be able to predict their clinical performance. 

The immersion assay performed in artificial saliva at different pH levels suggested that, irrespective of the pH value of the medium, the two investigated CAD/CAM milled FRCs (Trinia^TM^, Trilor) present similar trends in terms of mass variation after 21 days of immersion, suggesting their stability when in contact with artificial saliva, an aspect which was also highlighted by SEM analysis performed on the immersed surfaces.

Within the limitations of this in vitro study, it can be concluded that the novel composite biomaterials can be a proper alternative for metal alloys used for prosthetic frameworks and represent a viable option of dental materials for the oral rehabilitation of patients suffering from GERD.

## Figures and Tables

**Table 1:** Name, sample codification, and manufacturer of materials used.

Material Trade Name	Codification	Manufacturer
TriniaTM	C1	BICON Dental Implants (Boston, MA, USA)/https://www.trinia.com/ (Accessed on 10 October 2021) [21]
TriLor	C2	Bioloren SRL (Saronno, Italy)/https://bioloren.com/en/prodotti/trilor-en/trilor-disk/ (Accessed on 11 October 2021) [24]

**Table 2:** Chemical composition of Carter Brugirard artificial saliva.

Chemical Substances	Quantity/L (g)
Na_2_HPO_4_	0.19
NaCl	0.70
KSCN	0.33
KH_2_PO_4_	0.26
NaHCO_3_	1.50
Ureea	1.30

**Table 3:** Elemental composition of the samples obtained by EDS.

Sample	Spot	Element (wt.%)									
		O	C	Si	Ca	Al	Na	Mg	Ti	Cl	S
C1	1	44.54	31.12	11.97	6.7	4.67	0.43	0.33	0.24	-	-
	2	25.03	69.89	1.52	1.09	0.62	-	-	1.63	0.22	
C2	1	44.02	21.82	17.56	10.09	5.9	-	0.3	-	-	0.31
	2	27.05	55.59	3.43	7.69	1.11	-	-	2.73	0.1	2.29

**Table 4:** Mass evolution of the C1 and C2 samples after immersion in artificial saliva.

Carter Brugirard SA pH	Material	Mass Evolution (mg)			
		Day 3	Day 7	Day 14	Day 21
pH = 7.6	C1	−0.72 (±0.01)	−0.18 (±0.01)	0.51 (±0.01)	1.65 (±0.02)
	C2	−0.99 (±0.02)	−0.34 (±0.01)	0.54 (±0.01)	1.54 (±0.01)
pH = 5.7	C1	−0.73 (±0.01)	−0.27 (±0.01)	0.59 (±0.02)	1.72 (±0.02)
	C2	−1.11 (±0.02)	−0.40 (±0.01)	0.59 (±0.01)	1.82 (±0.02)
varying pH from 5.7 to 3	C1	−0.86 (±0.02)	−0.46 (±0.01)	0.30 (±0.02)	0.98 (±0.02)
	C2	−1.05 (±0.02)	−0.68 (±0.02)	0.18 (±0.01)	1.10 (±0.01)

## Data Availability

Data sharing is not applicable to this article.
